# A structure–function based approach to floc hierarchy and evidence for the non-fractal nature of natural sediment flocs

**DOI:** 10.1038/s41598-021-93302-9

**Published:** 2021-07-07

**Authors:** Kate L. Spencer, Jonathan A. T. Wheatland, Andrew J. Bushby, Simon J. Carr, Ian G. Droppo, Andrew J. Manning

**Affiliations:** 1 School of Geography, Queen Mary University of London, Mile End Road, London, E1 4NS UK; 2 School of Engineering & Materials Science, Queen Mary University of London, Mile End Road, London, E1 4NS UK; 3 Institute of Science, Natural Resources and Outdoor Studies, University of Cumbria, Ambleside, LA22 9BB Cumbria UK; 4 Environment and Climate Change Canada, 867 Lakehouse Road, P.O. Box 5050, Burlington, ON L7S 1A1 Canada; 5 HR Wallingford, Howbery Park, Wallingford, OX10 8BA Oxfordshire UK

**Keywords:** Hydrology, Physical oceanography

## Abstract

Natural sediment flocs are fragile, highly irregular, loosely bound aggregates comprising minerogenic and organic material. They contribute a major component of suspended sediment load and are critical for the fate and flux of sediment, carbon and pollutants in aquatic environments. Understanding their behaviour is essential to the sustainable management of waterways, fisheries and marine industries. For several decades, modelling approaches have utilised fractal mathematics and observations of two dimensional (2D) floc size distributions to infer levels of aggregation and predict their behaviour. Whilst this is a computationally simple solution, it is highly unlikely to reflect the complexity of natural sediment flocs and current models predicting fine sediment hydrodynamics are not efficient. Here, we show how new observations of fragile floc structures in three dimensions (3D) demonstrate unequivocally that natural flocs are non-fractal. We propose that floc hierarchy is based on observations of 3D structure and function rather than 2D size distribution. In contrast to fractal theory, our data indicate that flocs possess characteristics of emergent systems including non-linearity and scale-dependent feedbacks. These concepts and new data to quantify floc structures offer the opportunity to explore new emergence-based floc frameworks which better represent natural floc behaviour and could advance our predictive capacity.

## Introduction

Flocs are fragile, complex, low density aggregates of minerogenic and biogenic material with fluid-filled pore space^1,2^ and can represent the main component of suspended particulate matter (SPM) where sediment supply is dominated by fine-grained material (clay, silt and fine sand). SPM transport is critical to the fate and flux of sediment, carbon, nutrients, contaminants and pathogens through all natural aquatic environments. Therefore, understanding and predicting floc transport behaviour is essential for the sustainable management of our waterways, fisheries and marine industries.

Floc behaviour is dependent upon the size, shape, density and porosity of floc aggregates, and such data are critical input parameters for the mathematical models that predict fine sediment transport and flocculation. These inherently 3-dimensional (3D) characteristics are challenging to measure. Flocs are fragile, difficult to sample, and range in size across a spectrum from colloidal particles (nanometres) to larger aggregates (1000 s microns) spanning detection and resolution limits of multiple analytical techniques (transmission electron microscopy, scanning electron microscopy, confocal laser scanning microscopy, optical microscopy and video/image/laser analysis^3^. Consequently, critical parameters such as size and shape are frequently measured as 2-dimensional (2D) simplifications of complex 3D structures using e.g., image or laser analysis, whilst density and porosity are estimated indirectly from settling velocity and assuming spherical shape.

Given these limitations in the availability of observational data for floc characteristics, and to account for floc variability and derive a relationship between density, floc size and dynamic behaviour, flocs are considered to have fractal geometry^4^. The fractal-based model is mathematically and computationally simple, assumes that floc structures and properties are scale invariant, and is widely used to predict floc behaviour (e.g., settling velocity, rate of floc aggregation and disaggregation)^4–6^. Many studies report that primary particle properties (e.g., size and density) are the most sensitive parameters that control floc dynamics in a fractal model^6,7^. Such properties and the fractal dimension, which is usually derived in 2D^4^, are relatively simple to measure for mineralogically homogeneous or experimental sediments. A fractal dimension of 1 indicates near spherical, compact flocs, with larger values (up to 3) indicative of ‘looser’, more complex flocs. A single fractal dimension of around 2 is frequently used to solve the fractal analytical flocculation equation^8^. However, fractal dimension is strongly influenced by sediment composition, and in particular the presence of organic material where extracellular polymeric substances (EPS) can enhance the ‘stickiness’ of flocs^9^. Therefore, the application of fractal-based models becomes more challenging in compositionally variable natural aquatic environments where sediments have high organic matter, mixed mineralogy and microbial content. In addition, real flocs are multi-component with primary particles of different densities, and measuring real fractal dimensions is problematic.

Field and laboratory observations confirm that flocculation processes (e.g., flocculation rate or efficiency) and the fractal dimension of flocs formed can vary significantly depending on the types of primary particles (sediment composition) and environmental conditions (e.g. salinity or SPM concentration)^10–12^. Therefore, the relationship between floc size, density and hence settling velocity vary spatially and temporally^6,13^. Empirical formulations also suggest that the fractal dimension varies with floc size^11,14^. Unlike Verney et al.^13^ who utilise a floc diameter parameter, Maggi et al.^14^ describe floc population based on the number of primary particles in the flocs, which appears to make the incorporation of a variable fractal dimension straightforward. Moreover, Maggi et al.^14^ adopt a sophisticated collisional efficiency closure that considers the effects of floc size and permeability.

In response to this, increasingly many models relax the assumption that the fractal dimension is invariant with floc size ^7,11,15–17^ and assign variable fractal dimensions dependent on observations of floc size distribution. These models recognise that multiple levels of floc aggregation (or a floc ‘hierarchy’) must exist based on observations of multi-modal floc size distributions and hence represent flocculation processes using multiple class population balance equations (PBE) and variable fractal dimension, e.g., Eisma^18^, Manning and Dyer^19^ and Shen et al.^16^ simulate the representative sizes and mass fractions using multiple floc aggregation levels—microflocs, macroflocs and megaflocs.

The large variability of floc composition (e.g., mineral content, particle size and organic matter content), recognition that fractal dimension varies with size and composition, and the likelihood that different flocculation mechanisms are restricted to specific length scales, collectively all indicates that the structure of natural flocs cannot be self-similar^2,11,20–23^, and are ‘pseudo-fractal’ at best. Indeed, the fractal nature of natural sediment flocs is widely contested and is largely based on circumstantial evidence and the observed scaling of density (decreasing) and porosity (increasing) with increasing floc diameter. As a result, whilst current fractal-based flocculation models present a mathematically workable approach, they are still not fully robust or efficient^11,20–22,24^. The development of alternative frameworks is limited by the lack of direct, quantitative, observational data of floc structures and the mechanics of their development, and hence understanding of their behaviour in aquatic environments across the full floc size spectrum.

We have previously developed protocols to collect and stabilise flocs, and using correlative tomography, to observe and quantify 3D floc structures and characteristics from 10^1^ nm to 10^3^ µm scale^25–27^. Here, we show how these new observations of fragile floc 3D structures and particle interactions across all relevant length scales demonstrate unequivocally that natural flocs are non-fractal. We propose a floc hierarchy that is based on observations of 3D structure and function. We then discuss how our data indicate that flocs possess characteristics of emergent systems including non-linearity and scale-dependent feedbacks. These concepts and new data to quantify floc structures offer the opportunity to improve current approaches to understanding fine sediment behaviour and to explore new emergence-based floc frameworks which better represent natural floc behaviour and could enable the development of innovative mathematical models to predict real floc behaviour.

## Materials and methods

### Summary of the correlative workflow and sample collection

This study utilised a novel sampling and imaging workflow that facilitated the collection and stabilisation of floc samples, and acquisition and correlation of multi-scale, 3D floc datasets. Detailed methodology can be found in Wheatland et al.^26,27^. The workflow used a targeted approach, whereby entire flocs were initially characterised at the mm-scale using X-ray computed micro-tomography (X-ray CT) before more focused analysis of sub-micron scale internal composition and structure using 3D focused ion beam nanotomography (FIB-nt). The resulting 3D datasets were transformed into voxel-based (3D pixel) data volumes and composition phases (e.g., clay minerals, organic matter etc.) segmented for quantification and visualisation^28^. 3D volumetric microscopy was combined with high-resolution (pixel size, c. 5 nm^2^) 2D scanning transmission electron microscopy (STEM) imagery and energy dispersive spectroscopy (STEM-EDS) to enable the classification of floc components. Most floc components were identified based on their grey-scale value, size and shape, organic structures samples were treated with electron-dense stains and elemental spectra obtained using STEM-EDS to aid compositional identification^26^. Spatial registration of datasets applied at increasing magnification ensured precise spatial referencing of submerged regions of interest within the floc sample and the ability to correlate floc structures at different length scales from nm to mm, correlating not only internal floc structure but whole floc geometric characteristics.

The floc samples presented here were sampled from natural estuarine cohesive sediment (silty clays) collected from the Thames Estuary, UK.

### Floc stabilisation

For the stabilisation of fragile hydrated floc samples the protocol outlined in Wheatland et al.^26^ was followed. Flocs were first fixed in a buffered solution of 0.15 M sodium cacodylate (pH 7.4) containing 2.5% glutaraldehyde and 2% formaldehyde with 2 mM calcium chloride before being embedded in Durcupan, a hydrophobic resin. Intermediate steps were implemented to improve contrast and replace pore water with resin, achieved by the addition of heavy metal stains (uranyl acetate, thiocarbohydrazide and lead aspartate) and washing samples in an ethanol series (20%, 50%, 70%, 90%, 100%, 100%) followed by anhydrous acetone.

### 2D STEM imaging

Dark-field STEM imagery was obtained from the ultrathin-sections using an FEI Inspect-F field emission gun (FEG) SEM (Hillsboro, Oregon, USA) operating at 30 kV and fitted with a split detector STEM stage.

### 3D FIB-nt

Volumes were obtained using an FEI Quanta 3D FEG FIB-SEM (Hillsboro, Oregon, USA). For FIB-nt block preparation and data collection, the protocol of Bushby et al.^25,29^ was employed for the steps following sample preparation. Samples were inserted into the stage of the Quanta and raised to the eucentric height (10 mm) where the electron and (gallium) ion beams converge. With both beams focused on a coincident point on the sample surface, the stage was tilted to an angle of 52° to bring the trimmed block-face into a position perpendicular to the ion beam. This enabled precision milling and imaging of the exposed subsurface. Prior to serial sectioning a smooth, dense, protective platinum coating with a consistent thickness of ~ 1 µm, was deposited over the selected region of interest to minimise milling fluctuations that can result in morphological defects on the milled cross-section. The target volume was then isolated from the surrounding material by milling trenches on three sides of its perimeter to create a suitably sized cube. An accelerating voltage of 30 kV and a current of 0.5 – 5 nA for the ion beam was selected for milling. Once a cube was prepared the front trench was enlarged to reveal a cross-section for imaging and side trenches eroded to act as repositories into which sputtered material could collect. Serial sectioning was achieved using Auto Slice & View software (Thermo Fisher Scientific, https://www.thermofisher.com/uk/en/home/electron-microscopy/products/software-em-3d-vis/auto-slice-view-4-software.html) and an image sequence acquired using the backscattered signal operating at 3 kV and 4 nA electron beam. Slice thickness was adjusted to match that of the pixel size of the image area to ensure an isotropic voxel resolution (3D pixel). The regular spacing between individual image slices allows entire image sequences to be transformed directly into voxel-based (3D pixel) data volumes suitable for quantitative analysis.

### 3D X-ray CT

CT scans were performed using two Nikon Metrology (Tring, UK) XT H 225 microtomographs, one configured with a 225 kV tungsten reflection target and the other with a 180 kV tungsten transmission target. Medium-resolution imaging of entire flocs was conducted using the reflection target CT with a focal spot size of ~ 10 µm, whilst high-resolution imaging of smaller sub-regions was undertaken using the transmission target CT which had a spot size of ~ 1 µm. Both CT scanners were fitted with Perkin Elmer (Waltham, Massachusetts, USA) 16-bit flat-panel detectors. During data acquisition the source was operated at a voltage of 60–150 kV and a current of 50–160 µA. A 1 mm thick copper filter was used to absorb X-rays in the lower end of the energy spectrum, i.e. ‘soft’ X-ray with energies < 30 kV. The resulting raw X-ray projections represent differences in X-ray energy attenuation, related to material density and the absorbing material’s attenuation coefficient. Raw X-ray projections were reconstructed through tomographic back-projection within CT Pro 3D (Nikon, Tokyo, Japan) to yield 3D volumetric models. During reconstruction data artefacts resulting from beam hardening and the centre of rotation were also addressed.

### 3D visualisation of the correlative datasets

All image processing steps were conducted using the imaging software Fiji/ImageJ v2^30^ (https://imagej.net/Fiji). To address the inherent misalignment between consecutive images within FIB-nt stacks and remove artefacts associated with the drift of the electron beam or sample, an alignment algorithm was applied. Segmentation was performed using a semi-automated segmentation tool capable of machine learning, after which volumes were imported into Avizo v9.0 (Thermo Fisher Scientific) for landmark based registration. Supplementary volumetric renderings were produced within the software package Drishti v2.6.2 (https://github.com/nci/drishti) in which the 2D transfer function editor, in addition to colour and transparency settings, permitted specific properties (i.e., false colour and opacity) to be assigned to segmented materials.

## Results and discussion

**Figure 1:**
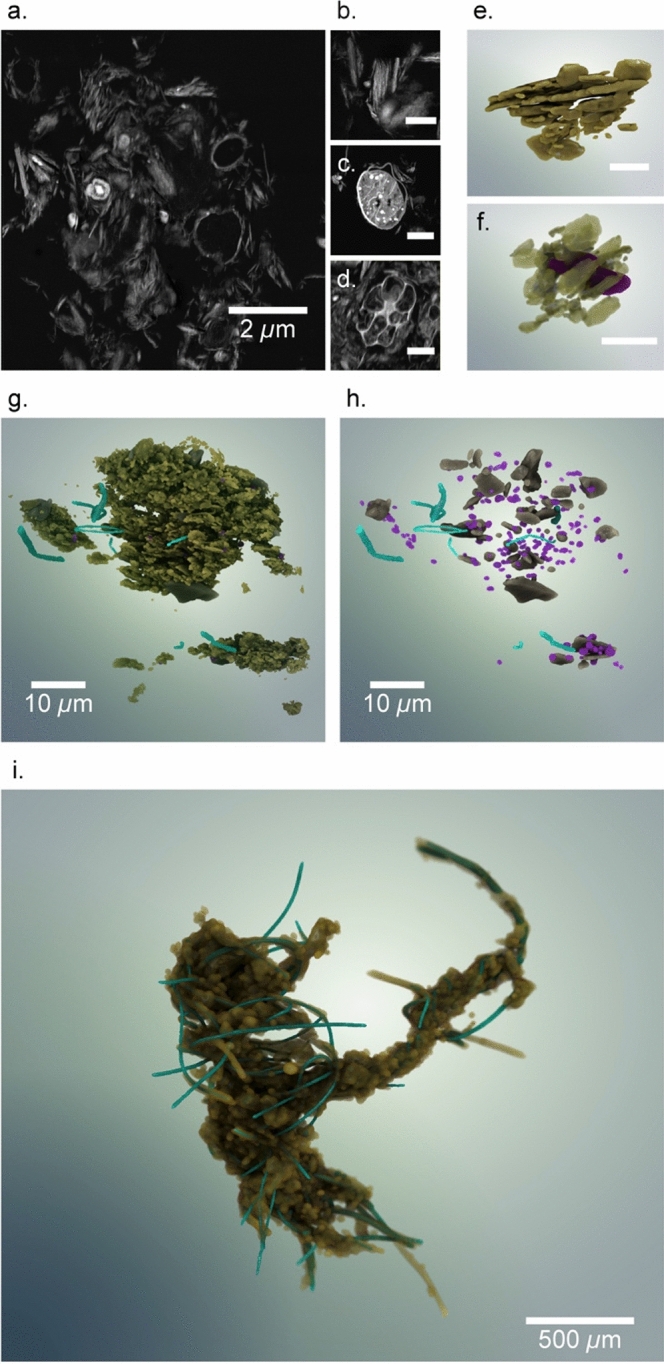
Image reconstructions of a natural floc sediment sample. 2D STEM images of a microfloc (**a**), a clay domain (**b**), a cyanobacteria associated with clay domains (**c**) and densely packed clay particles around a foraminifera (**d**). 3D FIB-nt reconstructions showing aligned multiple clay domains (**e**), clay domains arranged radially around a bacterial cell (false-coloured purple) (**f**), several microflocs (**g**) and the same sample but with clay minerals rendered transparent to show non-clay and bacterial components (**h**). A 3D X-ray CT reconstruction showing filamentous cyanobacteria (false-coloured blue) within mm-scale macrofloc structure (**i**). (**a**–**h** are high-resolution grey-scale image sequences obtained using FIB-nt; Scale bars—0.5 µm (**b**), 1 µm (**d**) and 2 µm (**c**, **e** and **f**)). Renderings of the 3D datasets (**e–i**) were generated using the software package Drishti v2.6.2 (https://github.com/nci/drishti). The figure was created using Adobe Illustrator CS6 (https://www.adobe.com/uk/).

Representative 2D and 3D correlative datasets showing spatially registered images from STEM, FIB-nt and X-ray CT are shown in Fig. 1. We have previously demonstrated the efficacy of our protocols to capture and stabilise delicate flocs without modification of floc geometry and with significant preservation of biological components^26^. Once segmented, these image data generate quantitative 3D reconstructions of particle–particle and nm to mm scale (3D FIB-nt and 3D X-ray CT respectively) structural associations and are false coloured to aid visualisation of complex composition. Flocs were characterised into functional groups (FG) according to observations of representative particle–particle interactions and the 3D spatial arrangement and association of floc components.

### FG-1—‘primary particles’

At the highest resolution, 2D dark-field STEM images of floc samples identify a wide range of primary particles typically found in natural fine sediment, including inorganic mineral particles (e.g., clays, silts and fine sands) and organic matter with a living (e.g., bacteria, diatoms etc.) and non-living component (e.g., detritus) (Fig. 1a–d). Identification of primary particles in STEM, through spatial registration allows their identification and segmentation in FIB-nt generating 3D datasets. Some materials, including clay minerals, are not found naturally as individual, dispersed particles, but instead form stable assemblages known as ‘domains’ consisting of multiple clay platelets aligned face-to-face^31^ (Fig. 1b). Primary particles are the smallest, indivisible constituents that function as distinct units, represent the simplest units in the ordered structure of floc aggregation^32^, and have been identified by many other workers using TEM approaches^1,33^.

### FG-2—‘particle–particle associations’

Particle interactions within turbulent aquatic environments are inevitable due to fluid hydrodynamics, and organisms actively searching to colonise surfaces for habitat development and food acquisition (i.e., attached dissolved organic carbon). These interactions can be observed in 2D and dark-field STEM imagery shows particle–particle associations including simple associations between multiple clay domains, and associations between dissimilar inorganic and organic floc components such as quartz grains, multiple clay domains and bacteria (e.g., Fig. 1; see also Supplementary Information for more examples). Again, such associations have been observed elsewhere^1,26,34,35^. The nature of these inter-particle interactions are dependent on the relative cohesive and adhesive properties of primary particles^36^ and reflect the environment in which flocs form. Figure 1 illustrates that these flocs are dominated by fine-grained cohesive clay minerals associated with the muddy, estuarine sediments of the Thames Estuary.

However, accurate characterisation of 3D particle geometry, particle–particle associations and particle arrangements can only truly be achieved with volumetric imaging at the nano- to micron-scale^25,28^. 3D FIB-nt floc data volumes are shown in Fig. 1e–h and reveal multiple floc constituents, including pore space, clay minerals, non-clay minerals, unicellular organisms (primarily prokaryotes), filamentous cyanobacteria, decaying organic detritus and organo-mineral debris. Smaller sub-volumes reveal clear 3D particle–particle arrangements and associations including simple associations between clay particles oriented face-to-face and/or edge-to-face and more complex associations between dissimilar particles, e.g., microbes and clay domains (Fig. 1e,f). The arrangement of these particles confirms the mechanisms of flocculation and provides new information on resultant internal floc structures.

Cohesion (e.g., electrochemical flocculation) is the key interaction operating between chemically similar particles such as clay minerals and is dependent upon clay mineralogy and the ionic strength of the solution^37^. In 3D, it becomes clear how densely packed these FG-2 units are. Minimal nanoscale porosity is visible and cannot be clearly resolved between clay particles, but has previously been estimated as < 10% of total floc porosity^26^. In Fig. 1f, we see a bacterium (false coloured purple) radially surrounded by clay domains. Here, adhesion (e.g., bioflocculation) dominates where extracellular polymeric substances (EPS) secreted by microorganisms adhere to the surfaces of clay particles^1^. The resolution of FIB-nt (10 nm)^28^ prevents the detection of EPS, but it can be observed in STEM filling the pore spaces between clay particles (Fig. 1a). It is therefore likely that polymer bridging also influences the development of the clay particle associations observed within these floc samples.

The mechanisms driving the development of these particle–particle associations are clearly scale-dependent. Cohesion can only operate over short distances between 10^1^ and 10^3^ nm. Cells become associated with clay particles which provide a source of nutrients and protection from predation^38^. The radial arrangement of clays around the cell optimises access to these resources, but individual cells can only interact with a finite number of clay domains before becoming isolated inhibiting further aggregation (Fig. 1f). These 3D arrangements and particle interactions result in a negative feedback whereby primary associations reach a threshold size primarily controlled by the nature of primary particles and the distance over which these mechanisms can operate, limiting the continued aggregation of FG-2 floc units. Therefore, the characteristics of FG-2 units are defined by strong electro- and bioflocculation mechanisms which result in radial structures and very low or near absent nanoporosity. These structures will form strong, compact, high density flocs with near spherical shape and high volumetric fractal dimension. For example, many researchers observe ‘flocculi’ which are resistant to disaggregation even in highly turbulent conditions, have a high fractal dimension and are considered to be the building blocks of larger floc aggregates^17,32,39^.

### FG-3 ‘Microflocs’

Indicative examples of larger structural floc units, characterised by greater complexity and heterogeneity, higher intra-floc porosity and looser structures are shown in both 2D STEM (Fig. 1a) and within the 3D FIB-nt volume (Fig. 1g). These FG-3 ‘microflocs’ are composed of mixtures of both individual FG-1 primary particles (e.g., silt grains and amorphous organic detritus) and FG-2 particle–particle associations. These units ranged in size from 5 – 40 μm with much higher 10^0^ micron-scale internal porosity, exhibiting open ‘card-house’ structures, while more ‘compact’ units (diameter, ~ 30 μm) comprised of densely packed clay minerals and occasional diatom frustules. Figure 1g shows several FG-3 units and it is possible to distinguish between FG-3 units based on inter-aggregate microporosity (Fig. 1g), which enables individual microfloc units to be isolated from one another. Figure 1h shows the same cluster of FG-3 units but with clay minerals rendered transparent to reveal the spatial arrangement of non-clay minerals, likely fine-grained quartz (FG-1), and microbes likely to be the centre of microbe-clay associations (FG-2) within the microfloc. The open structure of these microflocs indicates mechanisms other than electrochemical flocculation are important. Here, aggregation is predominantly enabled through the presence of soluble EPS via polymer bridging^33^ with weaker electrochemical forces having a secondary effect. Filamentous bacteria (false coloured blue) were observed to extend through the FG-3 volumes, and were frequently observed within these units. These units represent heterogeneous associations of primary particles (FG-1) and particle–particle (FG-2) associations confirming previous descriptions of microflocs^17,32^, but can be further characterised by highly variable, micron-scale intra-aggregate porosity and aggregation is facilitated by polymer bridging. Whilst, the high resolution and 3D nature of our data also allow the discrimination of inter-aggregate porosity, suggesting the association of multiple microfloc units.

### FG-4 and FG-5—macroflocs and megaflocs

Larger flocs (diameter > 10^2^ µm) are a critical contribution to mass settling flux in the environment^40^. X-ray CT fills a resolution gap between STEM and FIB-nt, and floc cameras and optical/laser techniques providing information on 3D size, shape and internal structures^27,41^. Figure 1i shows a complex, highly tortuous, highly irregular megafloc with intra-aggregate porosity c. 10^1^ µm (also see Supplementary information for animated 3D movie). A key component of this large floc is the presence of filamentous cyanobacteria (false-coloured blue).

**Figure 2:**
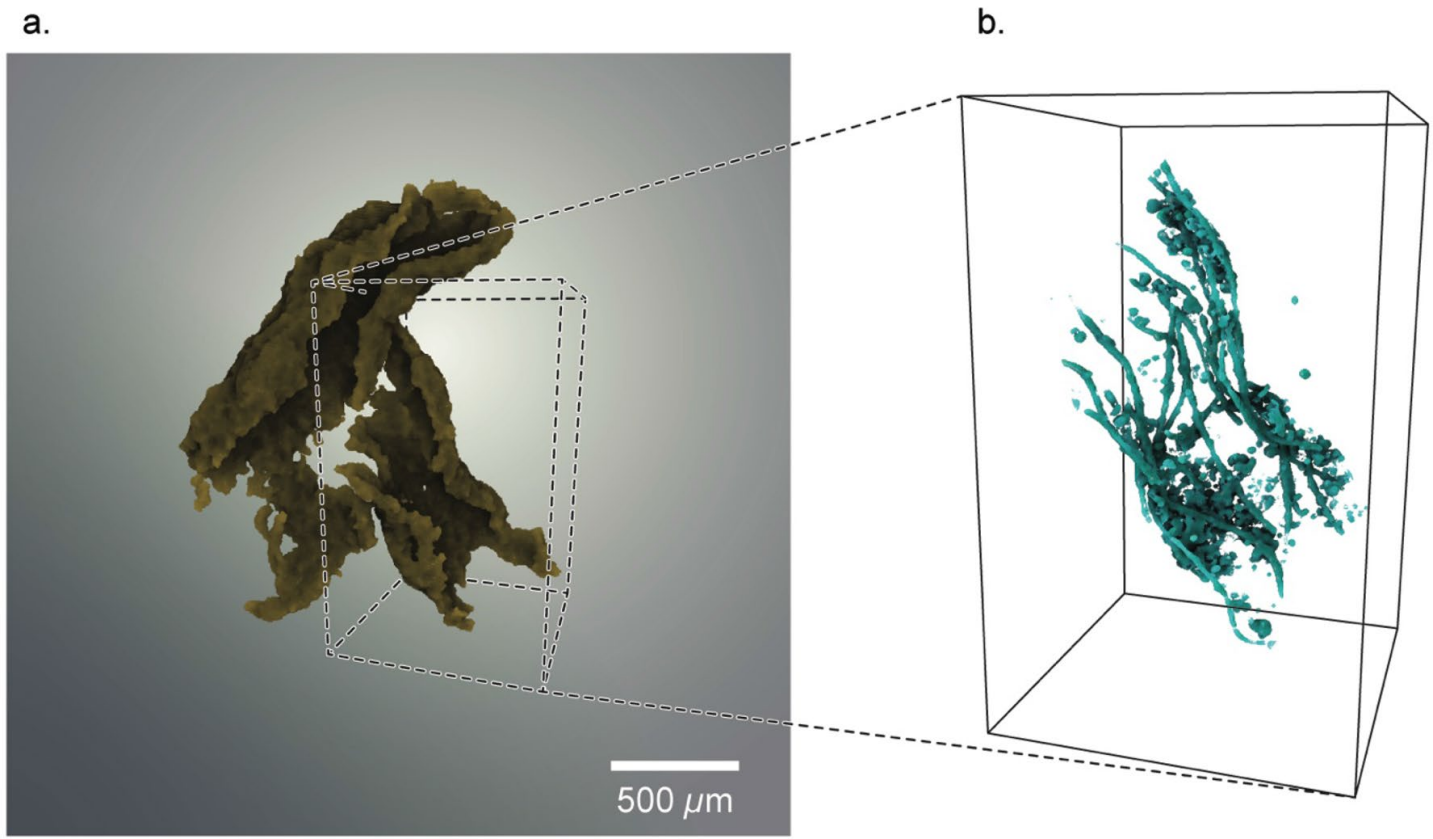
3D image reconstruction of a ‘macrofloc’ (FG-5) and the influence of filamentous microbes on floc structure. (**a**) 3D rendering of a ‘macrofloc’ (FG-5) clearly composed of smaller floc units (FG-4). (**b**) Sub-volume containing one of the FG-4 units identified in (**a**) but with minerogenic material render transparent to reveal the filamentous bacteria that act as a ‘backbone’ for the floc. Note the elongated, non-spherical shape of the floc which is directed by the filamentous morphology of the cyanobacteria. Protruding filamentous bacteria (PFB) act as ‘anchor points’ to join FG-4 units together (see **a**). 3D renderings were generated using Drishti v2.6.2 (https://github.com/nci/drishti). The figure was created using Adobe Illustrator CS6 (https://www.adobe.com/uk/).

The abundance and morphotype of bacteria influence the size and shape of these macro- and megaflocs. Cyanobacteria provide floc tensile strength, connectivity and flexibility, and anchoring mechanisms which enable the floc structures to respond to their dynamic environment^42–44^. Cyanobacteria also influence aggregation. Protruding filamentous bacteria (PFB) project beyond the floc periphery and link the FG-4 floc units at nodal ‘anchor’ points to create large FG-5 flocs (Fig. 1i). This dissipates turbulent eddies promoting further contact and linking floc units into larger entities (positive feedback). They can also act as a physical barrier preventing interaction with other floc structures limiting growth of FG-4 units (negative feedback)^44^. Alignment and orientation of the cyanobacteria (Fig. 2 and Supplementary Video) demonstrates their influence on floc shape resulting in an elongate ‘stringer’ type floc and as the cyanobacteria are broadly aligned there is a higher probability of anchor points towards the extremities of the FG-4 units, which results in FG-5 composed of multiple, connected FG-4 units. This additional level of structure incorporates highly tortuous, c. mm-scale porosity (Fig. 1i and Supplementary Video) which has the potential to create drag, influence settling behaviour^45^ and results in highly non-spherical aggregates.

Therefore, at the gross ‘whole floc’ scale, 3D structural data provides the evidence supporting the assertion that fractal dimension varies with higher level aggregation, and aggregation mechanisms and composition have a strong control on floc size and morphology.

## Floc hierarchy, non-fractal structures and flocs as emergent phenomena

**Figure 3:**
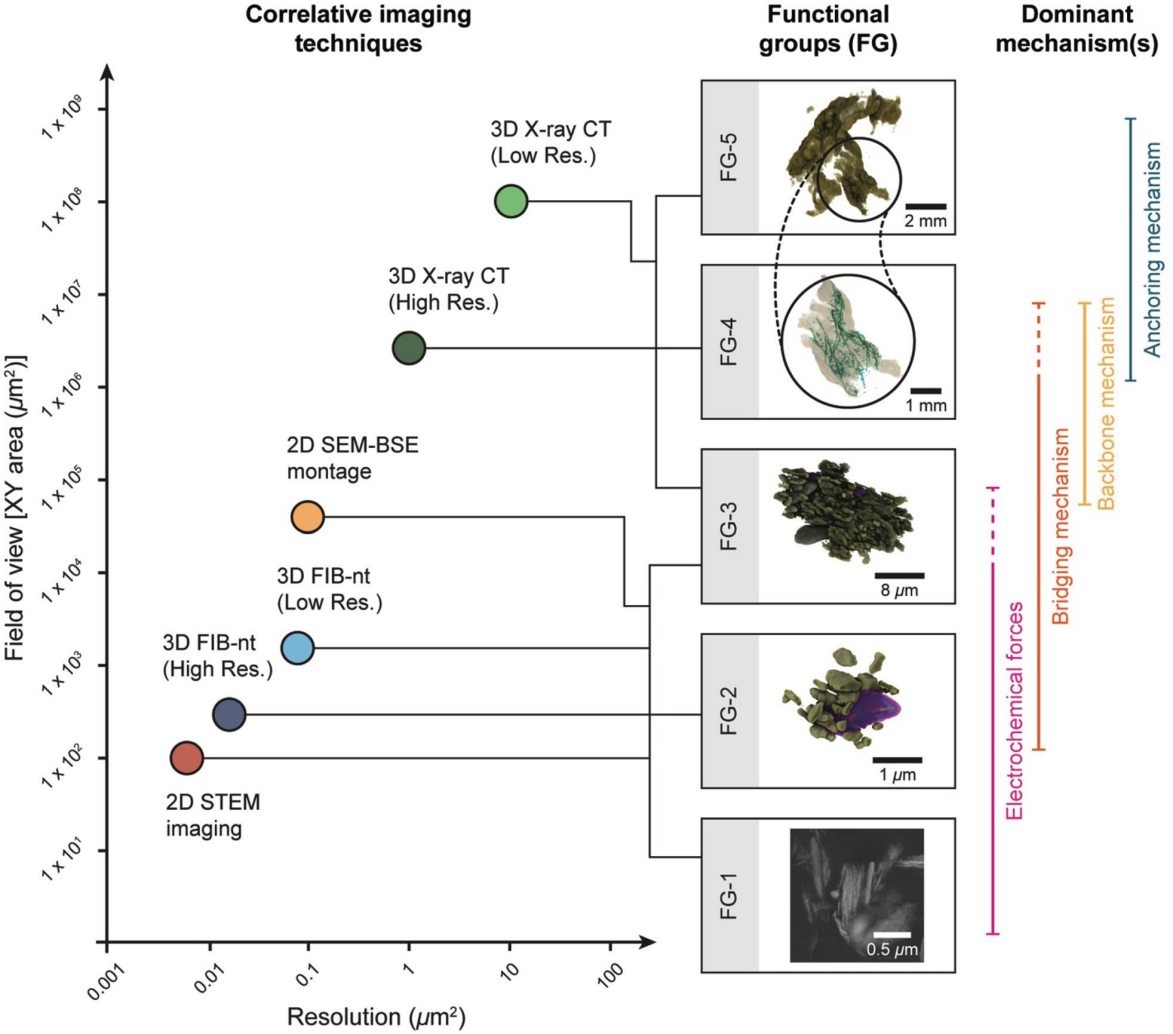
3D visualisation of representative natural flocs showing the functional groups (FG) that occur alongside the mechanisms that promote their formation. The correlative imaging techniques required to identify these particle–particle and structural associations are plotted by their resolution and field of view (XY). The figure was created using Adobe Illustrator CS6 (https://www.adobe.com/uk/).

Five functional groups can be characterised by the mechanisms of particle–particle interaction, the nature and size of intra- and inter-floc porosity and the 3D structural associations and spatial arrangement of floc components. Figure 3 shows these FGs, the imaging techniques used to observe them and the dominant scale-dependent binding mechanisms that define them. Mechanisms that promote aggregation, such as cohesion and bioflocculation, are scale dependent and result in indicative size limited 3D radial and close-packed structures (e.g., association between bacteria and clays), operate over multiple levels of aggregation and control floc size through both negative and positive feedbacks enabling flocs to reach adaptive equilibrium with the surrounding environment. In addition, key structural characteristics that can be observed here, such as porosity and floc shape also vary with scale controlled by both the nature of particle–particle interactions and floc composition, For example, porosity varies in size and morphology from simple, nanoscale pore space in FG-2 to complex, tortuous and highly irregular micron to millimetre scales pores and pore networks in FG-4 and FG-5, whilst floc shape varies from simple, near spherical FG-2 flocs to complex, irregular FG-5 units. This provides unequivocal evidence that natural flocs are non-fractal.

Previous studies have observed multiple modalities within floc size distributions (FSD) and used these observations to develop conceptual, multi-level aggregation models comprising: primary particles, flocculi, microflocs, macroflocs and megaflocs^3,16,17^. These images of real natural flocs confirm many of the assumptions of aggregation mechanism. Observations of FSD have been used to develop multi-class PBEs in flocculation and transport models. Significant variation exists over the number of modal classes (up to 4) and which geometric size ranges most accurately represent floc populations. In particular, there is a focus on defining a static boundary between floc ‘building blocks’ and larger, more fragile flocs, with the boundary ranging from e.g., between 120 and 200 microns^16,46^. To confuse matters further, this boundary describes the division between both micro and macroflocs, and macro and megaflocs, depending on the number of size classifications used. Here, we present the evidence defining microflocs (FG-3) structurally and functionally as a collection of primary particles (FG-1) and particle–particle associations (FG-2), with loose µm-scale intra-aggregate porosity, where aggregation mechanisms are dominated by electro-flocculation and polymer bridging. In contrast, macroflocs (FG-4) are defined by the importance of bacterial populations which exert strong control on floc aggregation mechanisms, shape and size of the floc units produced and result in large, tortuous pore channels through floc units and highly irregular floc shapes. Such large pore networks and irregular shapes are likely to have a significant impact on dynamic behaviour and demonstrate the strong influence that the microbiological community could have on fine sediment behaviour.

These functional groups operate over multiple, overlapping geometric scales and indicate that the use of modal classes, each with an assigned 2D fractal dimension, will never truly represent or predict the behaviour of natural flocs in the environment. Despite this critical flaw, suitable alternatives remain elusive due to the absence of observations and quantitative data on floc structures and particle–particle interactions. Emergence describes how complex systems arise from their underlying constituent components, exhibiting unexpected patterns, functions and behaviours that cannot be predicted from an understanding of the individual system constituents^47,48^ The evidence presented here indicates that flocs may possess many of the characteristics of emergent systems, which can be distinguished from other self-assembly behaviour. This includes non-linearity, scale-dependent feedback and interactions and self-regulating, adaptive equilibrium behaviour^47^. For example, we have evidence for both negative and positive feedbacks across spatial scales and we observe that interactions with bacteria may have both negative and positive feedbacks at different scales (creating both turbulence and promoting interaction, and protecting against further aggregation).

An emergence approach has successfully been used to explain and predict patterns and behaviour in many natural systems^49,50^ and provides an alternative, potentially more realistic approach for representing real multi-component floc structures with complex non-linear dynamics. Such mathematical process-based models require data which capture quantitative information to describe and explain feedbacks, interactions and spatial relationships (structures) between constituent components at all spatial scales^51^. This is in stark contrast to fractal-based approaches which are based on static dimensional or geometric data describing bulk floc characteristics (e.g., size, fractal dimension, density). The data generated here provide this quantitative information on floc structures and particle–particle interactions. For example, we have already demonstrated the potential to quantify accurately floc constituents e.g., pore space and bacterial counts^26^ and it will be possible (although beyond the scope of this study) to develop algorithms and machine learning approaches to quantify spatial organisation of floc constituents (including pores), and to characterise interactions (in terms of nature and scale). Therefore, these new 3D data present the opportunity to inform a new generation of emergent models. Utilising an emergence framework could better account for the spatio-temporal variations observed in natural sediment floc behaviour and provide a level of error checks that are not supported by current fractal approaches.

## Potential applications of 3D data to existing approaches

The scanning, data processing and visualisation approaches used here to generate fully rendered 3D images are extremely operator- and computer processor-intensive. Therefore, our approach is not suitable for application as part of e.g., a field monitoring campaign. However, whilst only a few indicative 3D images have been presented here, quantitative 3D data for 100–1000 s of individual flocs were generated, particularly for the X-ray CT analysis. These 3D data provide new opportunities to quantify accurately bulk properties of flocs including floc size and porosity (measured as occupied volume)^26,41^, shape (aspect ratio, sphericity and algorithms to quantify shape complexity), and 3D fractal dimension, and to semi-quantify density^41^. In addition, new mathematical approaches can be explored to quantify structural properties of flocs such as the size and connectivity of pore space to inform on the influence of drag and friction as fluid moves through a floc structure, and the spatial arrangement and distribution of individual floc components. Coupling these observations in the laboratory to measures of floc and fine sediment behaviour (e.g., settling velocity and erodibility) could improve our understanding of the controls on floc behaviour and the parameterisation of existing fine sediment transport models. This will be a major advancement as models are currently parameterised using data generated in 2D, or approximations (porosity and density) assuming flocs have spherical shape.

## Conclusions

- Assumptions of fractal geometry are currently used to aid the mathematical modelling of suspended sediment dynamics. Yet, it is widely recognised that this does not accurately represent natural floc systems.
- New quantitative 3D data provide unequivocal evidence to demonstrate that natural sediment flocs are non-fractal and that particle composition and inter-particle interactions have a strong influence on aggregate morphology.
- We propose an alternative five-level hierarchy of floc aggregation based on structure–function relationships rather than observations of geometric floc size distributions.
- New multi-scale, high resolution 3D data indicate that flocs possess characteristics of emergent phenomena including non-linearity, scale-dependent feedbacks and self-regulation. This offers the opportunity to explore new emergence-based floc frameworks and process-based models based on quantification of particle–particle interactions which better represent natural floc behaviour.

## Supplementary information

Supplementary Information 1.

Supplementary Figure 1.

Supplementary Video 1.

## Data Availability

Data are currently under embargo by the Natural Environment Research Council, but data will be made freely available on their data repository.
