# Supplementary material for: A structure–function based approach to floc hierarchy and evidence for the non-fractal nature of natural sediment flocs
Source: Sci Rep. 2021 Jul 7;11:14012. doi: 10.1038/s41598-021-93302-9 (PMC8263784; doi:10.1038/s41598-021-93302-9)
Supplement: Supplementary file 1 — Supplementary Information 1. [file 41598_2021_93302_MOESM1_ESM.docx]

**A structure-function based approach to floc hierarchy and evidence for the non-fractal nature of natural sediment flocs.**

Kate L. Spencer, Jonathan A.T. Wheatland, Andrew J. Bushby, Simon J. Carr, Ian G. Droppo and Andrew J Manning.

**Supplementary information**

**Movie**

3D reconstruction of a natural sediment floc. Filamentous cyanobacteria (blue) run through the floc and have a strong influence on shape. This can be seen clearly as the inorganic material (brown) in rendered transparent. The movie was created using Drishti v2.6 ([https://github.com/nci/drishti](https://eur01.safelinks.protection.outlook.com/?url=https%3A%2F%2Fgithub.com%2Fnci%2Fdrishti&data=04%7C01%7C%7C942677a7a3aa4e79b09e08d925df20d4%7C569df091b01340e386eebd9cb9e25814%7C0%7C0%7C637582465041118377%7CUnknown%7CTWFpbGZsb3d8eyJWIjoiMC4wLjAwMDAiLCJQIjoiV2luMzIiLCJBTiI6Ik1haWwiLCJXVCI6Mn0%3D%7C1000&sdata=%2Bbw3qjybFTZYg1VjMd%2BUy8%2FiDaRD7Rev66WOGMoGo7w%3D&reserved=0))

**Supplementary information Figure 1**

Dark-field STEM imagery of the types of primary associations (FG-2) observed within flocs. (**a**) Simple face-to-face association consisting of stacked clay domains (FG-1). (**b**) Clay domains arranged around a central bacterial cell. (**c**) Clay domains (FG-1) arranged around multiple bacterial cells. (**d**) EPS. (**e**) A large quartz grain with compacted clay domains adhered to its surface. (**f**) Closely associated clay domains likely originating from resuspended (consolidated) bottom sediment. The figure was created in the illustration software Adobe Illustrator CS6 (<https://www.adobe.com/uk/>).
