# Supplementary figures and images for: A structure–function based approach to floc hierarchy and evidence for the non-fractal nature of natural sediment flocs
Source: Sci Rep. 2021 Jul 7;11:14012. doi: 10.1038/s41598-021-93302-9 (PMC8263784; doi:10.1038/s41598-021-93302-9)

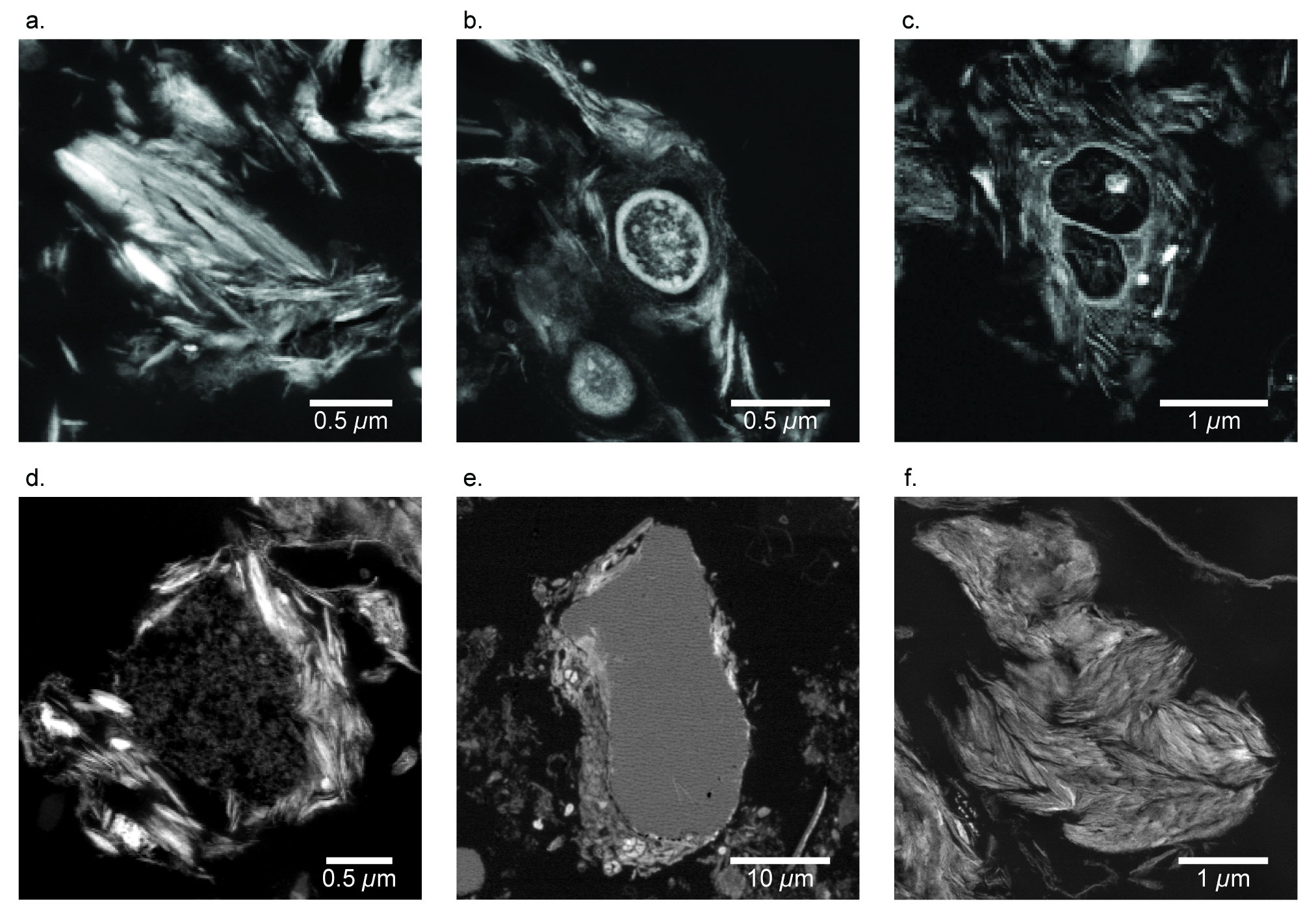

Supplement: Supplementary file 2 — Supplementary Figure 1. [file 41598_2021_93302_MOESM2_ESM.jpg]
